# Deep Learning Models for Predicting Malignancy Risk in CT-Detected Pulmonary Nodules: A Systematic Review and Meta-analysis

**DOI:** 10.1007/s00408-024-00706-1

**Published:** 2024-05-23

**Authors:** Wahyu Wulaningsih, Carmela Villamaria, Abdullah Akram, Janella Benemile, Filippo Croce, Johnathan Watkins

**Affiliations:** 1 The Royal Marsden, London, UK; 2 Faculty of Life Sciences & Medicine, King’s College London, London, UK; 3 Modamast Pte Ltd, Singapore, Singapore; 4 University Hospital of Wales, Cardiff, UK; 5 Optellum Ltd, Oxford, UK

**Keywords:** Pulmonary nodules, Lung cancer, Screening, Diagnosis, Chest CT

## Abstract

**Background:**

There has been growing interest in using artificial intelligence/deep learning (DL) to help diagnose prevalent diseases earlier. In this study we sought to survey the landscape of externally validated DL-based computer-aided diagnostic (CADx) models, and assess their diagnostic performance for predicting the risk of malignancy in computed tomography (CT)-detected pulmonary nodules.

**Methods:**

An electronic search was performed in four databases (from inception to 10 August 2023). Studies were eligible if they were peer-reviewed experimental or observational articles comparing the diagnostic performance of externally validated DL-based CADx models with models widely used in clinical practice to predict the risk of malignancy. A bivariate random-effect approach for the meta-analysis on the included studies was used.

**Results:**

Seventeen studies were included, comprising 8553 participants and 9884 nodules. Pooled analyses showed DL-based CADx models were 11.6% more sensitive than physician judgement alone, and 14.5% more than clinical risk models alone. They had a similar pooled specificity to physician judgement alone [0.77 (95% CI 0.68–0.84) *v* 0.81 (95% CI 0.71–0.88)], and were 7.4% more specific than clinical risk models alone. They had superior pooled areas under the receiver operating curve (AUC), with relative pooled AUCs of 1.03 (95% CI 1.00–1.07) and 1.10 (95% CI 1.07–1.13) *versus* physician judgement and clinical risk models alone, respectively.

**Conclusion:**

DL-based models are already used in clinical practice in certain settings for nodule management. Our results show their diagnostic performance potentially justifies wider, more routine deployment alongside experienced physician readers to help inform multidisciplinary team decision-making.

**Supplementary Information:**

The online version contains supplementary material available at 10.1007/s00408-024-00706-1.

## Introduction

Five-year US survival rates for lung cancer fall from 73% at stage I to 9–13% at stages IIIB and IV. [1] Hence, early diagnosis is critical to reducing mortality. Pulmonary nodules are often the first sign. [2] Around 5% of nodules 4–30 mm in size are malignant [3]. Nodules, benign and malignant, are detected in 1.6 million people in the US annually, [3] and the majority are detected in CT scans. [4]

These facts combine to establish two conclusions: (a) early detection and discrimination of pulmonary nodules are crucial to reducing lung cancer mortality, and (b) CT scans are an essential modality for this detection and discrimination.

However, discriminating between malignant and benign nodules is difficult. Assessing the risk of indeterminate nodules poses a particular challenge [5]. Clinical risk models, such as Herder, Mayo, and Brock [6–8], that use clinico-demographic or radiological inputs to aid physicians are commonly used.

Recently, image-based computer-aided diagnostic (CADx) models using deep learning (DL) have emerged to assess malignancy. These models are easy and fast to use *versus* clinical risk models. Therefore, image-based DL models have the potential to fulfil an unmet clinical-management need, [9] providing they produce comparable diagnostic performance.

The objective of this study was to assess the diagnostic performance of DL-based models for predicting the risk of malignancy in CT-detected pulmonary nodules. This is the first systematic review to provide a pooled analysis of studies that externally validate DL-based models and directly compare them with methods routinely used in clinical practice.

## Methods

### Search Strategy and Screening

An electronic search was performed in MEDLINE (PubMed), EMBASE, Science Citation Index, and Cochrane Library databases (from inception to 10 August 2023). Studies were deemed eligible if they were peer-reviewed experimental or observational articles that assessed the diagnostic performance of externally validated DL-based CADx models to predict the risk of malignancy in solid or part-solid nodules. The full set of keyword search terms (eTable 1) and selection criteria (eTable 2) are found in the Supplementary Material. References of key studies and domain-related systematic reviews were also investigated. This study followed PRISMA and Meta-analysis of Observational Studies in Epidemiology (MOOSE) guidelines. [10, 11]

**Fig. 1:**
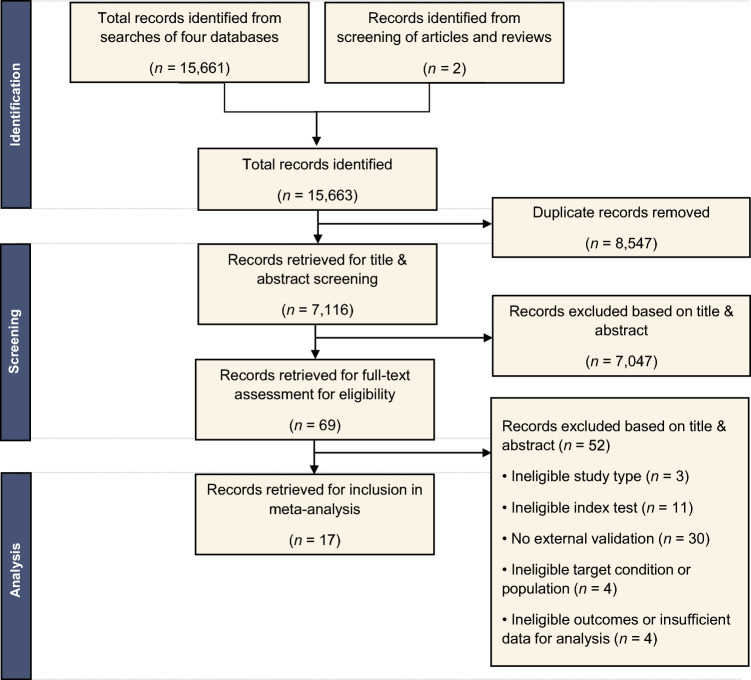
Literature search flow diagram

After removing duplicates, 7116 studies were found (Fig. 1). Screening out ineligible studies by title and abstract left 69 studies for final screening. Two investigators independently reviewed each text.

### Data Extraction and Quality Assessment

Information from included studies were extracted independently by two investigators (eTable 3). The data were subsequently checked against selection criteria (eTable 2). Risk of bias and applicability were independently assessed using the Quality Assessment of Diagnostic Studies 2 (QUADAS-2) tool [12].

### Statistical Analysis and Quantitative Synthesis

A meta-analysis of all included studies was conducted. For each index test type (DL-based models; physician judgement alone; clinical risk models alone; Lung-RADS-based models alone), pooled estimates of area under the curve (AUC), sensitivity, and specificity were calculated using a bivariate, random-effects approach. Deeks’ funnels were plotted to identify publication bias. To assess heterogeneity and inconsistency among the studies, *τ*^2^ statistic and *I*^2^ index values were calculated.

Potential sources of heterogeneity were investigated by conducting sub-group analyses, stratified by prevalence, route of detection, and geography.

Review Manager (RevMan) version 5.4, R statistical software version 4.3.1 (Beagle Scouts), and R packages ‘mada’ version 0.5.11 and ‘metafor’ version 4.2-0, were used to conduct the statistical analyses [13, 14].

## Results

### Study Characteristics

The literature search identified 17 studies for inclusion (Fig. 1), comprising 35 validation datasets, 8553 participants, and 9884 pulmonary nodules. Of these nodules, 1991 were confirmed to be malignant within the follow-up period (on average, 24 months) (Table 1).

**Table 1:** Characteristics of included studies

ID	Study	Country	Deep learning-based index test	Non-deep learning-based index test	Number of participants in validation	Number of nodules or images in validation	Number of malignant nodules or images (prevalence)^a^	Type of CT scan by chronology	Route of detection	Relevant outcomes reported^b^
01	Adams et al. [25]^,c^	USA	DL CNN (Google AI)	Physician readers (6 radiologists)	192 (3.197 weighted)	192 (3197 weighted)	35 (18%)	Initial	Screening	Sensitivity Specificity
02	Adams et al. [26]^,c^	USA Canada	RevealAI-Lung supervised ML classifier (mSI) and Lung-RADS	LUNG-RADS criteria retrospectively applied	306	486	95 (20%)	Initial	Screening; Incidental	Sensitivity Specificity
03	Ardila et al. [27]	USA	DL CNN (Google AI)	LUNG-RADS criteria retrospectively applied; Physician readers (6 radiologists)	815	815	123 (15%)	Initial	Screening	Sensitivity Specificity AUC
04	Baldwin et al. [22]	UK	DL CNN model (LCP)	Brock model	1187	1397	234 (17%)	Initial	Incidental	Sensitivity Specificity AUC
05	Chen et al. [15]	China South Korea	DL non-CNN XGBoost-based model (PKU-M)	Brock model Mayo model Physician readers (radiologist and 3 thoracic surgeons)	298	783	444 (57%)	Initial	Incidental	Sensitivity Specificity AUC
06	Chen et al. [28]	China	DL CNN model (Deepwise Healthcare)	Physician readers (2 radiologists)	104	148	85 (57%)	Initial	Incidental	Sensitivity Specificity
07	Çoruh et al. [29]	Turkey	DL CNN model	Physician readers (2 radiologists)	158	158	77 (49%)	Initial	Incidental	Sensitivity Specificity
08	Gao et al. [30]	USA	Co-learning model fed by image-based non-CNN DL model and CDE	PLCO_M2012_ Brock model	147	220	40 (18%)	Initial	Screening	AUC
09	Gao et al. [31]	USA	Co-learning model fed by image-based non-CNN DL model, biomarkers, and CDE (M3Net)	Mayo model Brock model	387	387	197 (51%)	Initial	Incidental	AUC
10	Huang et al. [24]	Canada	DL MLP model (DeepLR)	LUNG-RADS criteria retrospectively applied	2294	2294	92 (4%)	Follow-up	Screening	Sensitivity Specificity AUC
11	Hunter et al. [21]^,c^	UK	DL non-CNN model fed by radiomic feature selection (LN-RPV)	Physician readers (3 radiologists) Brock model Herder model	147	151	23 (15%)	Initial	Incidental	Sensitivity Specificity AUC
12	Jacobs et al. [32]	USA Denmark Canada	DL CNN model (grt123; Liao et al. [33] DL CNN model (JWDH) DL CNN model (Aidence)	Physician readers (11 radiologists)	150	150	50 (33%)	Initial	Screening	AUC
13	Kim et al. [17]	USA UK	DL CNN model (LCP)	Physician readers (6 radiologists) Physician readers (6 pulmonologists)	300	300	150 (50%)	Initial	Screening; Incidental	Sensitivity Specificity AUC
14	Liu et al. [34]	China	DL CNN model	Physician readers (6 radiologists)	153	168	112 (67%)	Initial	Incidental	Sensitivity Specificity AUC
15	Massion et al. [16]	USA UK	DL CNN model (LCP)	Brock model Mayo model	543	579	127 (22%)	Initial	Incidental	Sensitivity Specificity AUC
16	Trajanovski et al. [35]	USA	DL CNN model (N-Net)	Brock model Physician readers (6 radiologists)	773	773	42 (5%)	Initial	Screening	Sensitivity Specificity AUC
17	Venkadesh et al. [36]	USA Netherlands Belgium	DL CNN model	Brock model Physician readers (11 physicians)	599	883	65 (7%)	Initial	Screening	Sensitivity Specificity AUC

*CNN* convolutional neural network, *ML* machine learning, *CDE* clinical data elements, *MLP* multilayer perceptron

^a^Average across a study’s dataset

^b^A few studies reported outcomes in addition to sensitivity, specificity, and AUC

^c^DL-based model validated in an external dataset, but not directly compared to models or methods in clinical use. Direct comparison with physician readers and clinical risk models (Brock and Herder) was performed on an internal validation dataset (i.e. taken from the same cohort—but not the same data—as the model’s training dataset)

All the studies’ datasets save one, were retrospective cohorts, with one study containing a prospective-cohort dataset [15]. Datasets included populations from North America (11 studies), Europe (six studies), and Asia (four studies) (Table 1).

Studies primarily assessed diagnostic performance. Some studies reported clinical utility outcomes, such as diagnostic re-classification [16, 17]. However, due to inconsistency, it was not possible to conduct a meta-analysis on clinical utility. The main outcomes sought were the confusion matrices, sensitivity and specificity, and AUCs (Table 1). Many studies did not report confusion matrix values directly. As such, these were calculated using reported sensitivity, specificity, and prevalence values.

Sixteen DL-based CADx models were identified from the included studies. The commonest learning algorithm used were convolutional neural networks (CNNs). Ten of the 16 models and 11 of the 17 included studies used a CNN algorithm as the basis for their malignancy prediction score.

For the external validation index tests, the commonest comparator was physician readers (13 of 35 datasets, from 11 studies). The majority were radiologists with ≥ 3 years’ experience.

The Brock model was the commonest clinical risk model (12 datasets from eight studies), followed by the Mayo model with eight datasets from three studies. The Mayo model is considered the most externally validated model [18], but the Brock model performs better in screening populations [19, 20].

Most studies considered participants in the 50–75 age bracket. All studies included both female and male participants. The studies spanned the range of nodule sizes [21].

The average prevalence of malignancy across studies was 23%. Most incidentally detected nodules had prevalence ≥ 20%, whereas most screening populations had prevalence < 20%.

### Diagnostic Performance

#### DL-Based Models

**Fig. 2:**
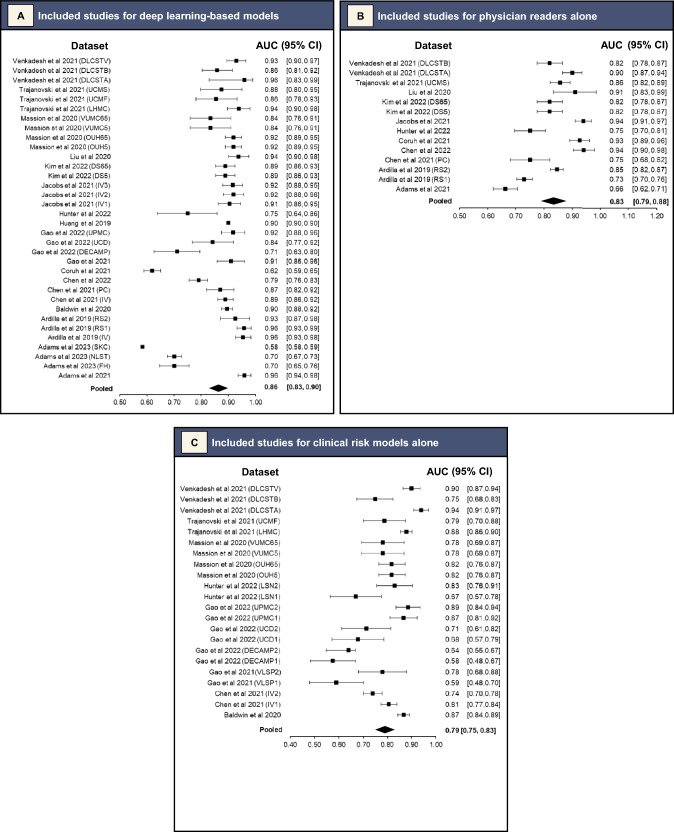
Pooled areas under the curve (AUC) analyses of the included studies and their datasets

**Fig. 3:**
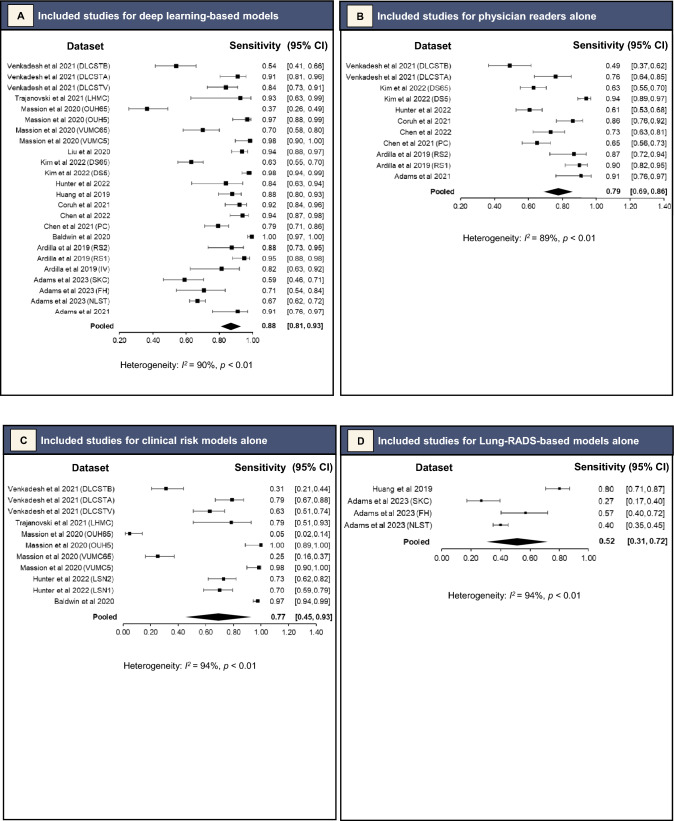
Pooled sensitivity analyses of the included studies and their datasets

**Fig. 4:**
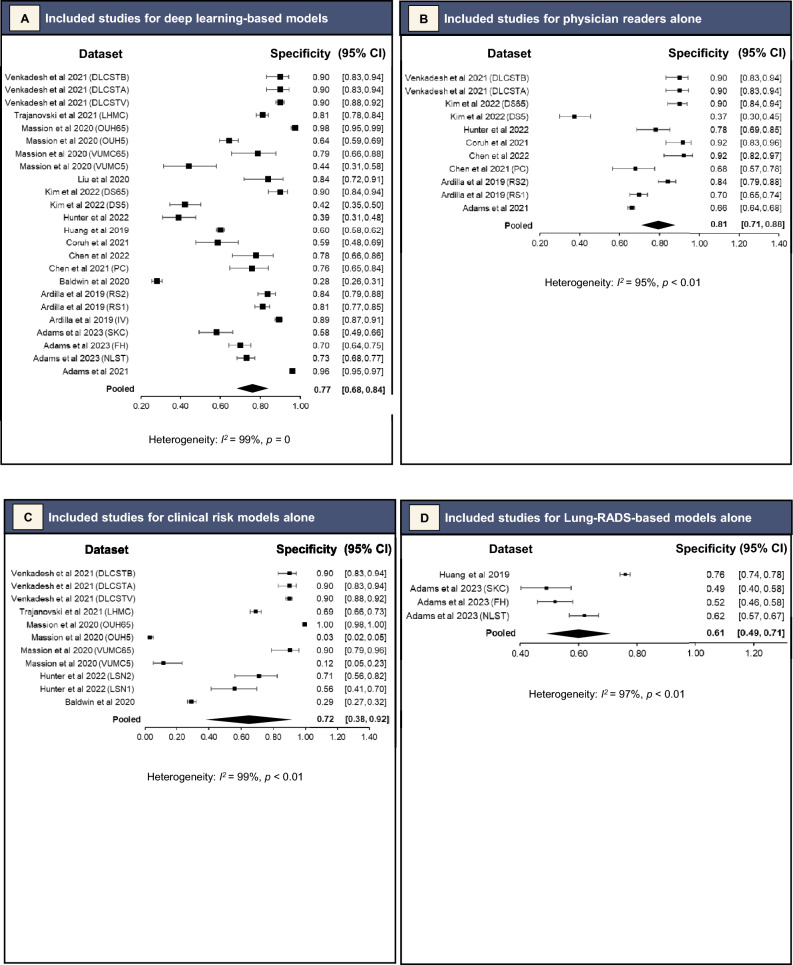
Pooled specificity analyses of the included studies and their datasets

For the DL-based models, meta-analysis of 34 datasets that reported AUC values or for which AUC values were able to be derived gave a pooled AUC of 0.86 (95% CI 0.83–0.90). (Fig. 2A). Sensitivity ranged from 0.37 (95% CI 0.25–0.50) for a 0.98 (95% CI 0.95–0.99) specificity [16], to 1.00 (95% CI 0.98–1.00) for a 0.28 (95% CI 0.26–0.31) specificity (Figs. 3A and 4A, respectively) [22]. Meta-analysis of 24 datasets gave a pooled sensitivity of 0.88 (95% CI 0.81–0.93) and specificity of 0.77 (95% CI 0.68–0.84) (Figs. 3A and 4A, respectively).

They had an *I*^2^ index of 90% (*p* < 0.01) for sensitivity and 99% (*p* = 0) for specificity, corresponding to very high statistical heterogeneity (an *I*^2^ value ≥ 75% was indicative of heterogeneity). The Deeks’ funnel plot showed no significant asymmetry, with *p* = 0.08 (a *p* < 0.05 result was assumed to be statistically significant), indicating no evidence of publication bias (eFigure 1A).

#### Physician Readers Alone

Separate pooled analysis for physician readers gave a pooled AUC slightly lower than DL-based models at 0.83 (95% CI 0.79–0.88) (Fig. 2B). They had sensitivity of 0.79 (95% CI 0.69–0.86) and specificity of 0.81 (95% CI 0.71–0.88) (Figs. 3B and 4B, respectively). Their *I*^2^ index was 89% (*p* < 0.01) for sensitivity and 95% (*p* < 0.01) for specificity, demonstrating high statistical heterogeneity. The Deeks’ funnel plot showed no significant asymmetry, with *p* = 0.31, indicating no evidence of publication bias (eFigure 1B).

#### Clinical Risk Models Alone

Pooled analysis for clinical risk models gave pooled AUC of 0.79 (95% CI 0.75–0.83) (Fig. 2C). They had sensitivity of 0.77 (95% CI 0.45–0.93) and specificity of 0.72 (95% CI 0.38–0.91) (Figs. 3C and 4C, respectively). Their *I*^2^ index was 94% (*p* < 0.01) for sensitivity and 99% (*p* < 0.01) for specificity, demonstrating very high statistical heterogeneity. The Deeks’ funnel plot showed no significant asymmetry, with *p* = 0.28, indicating no evidence of publication bias (eFigure 1C).

#### Lung-RADS-Based Models

Lastly, pooled analysis for Lung-RADS-based models gave a pooled sensitivity of 0.52 (95% CI 0.31–0.72) (Fig. 3D), and specificity of 0.61 (95% CI 0.49–0.71) (Fig. 4D). They had an *I*^2^ index of 94% (*p* < 0.01) for sensitivity and 97% (*p* < 0.01), demonstrating very high statistical heterogeneity. There were insufficient studies for a Deeks’ test.

#### Sub-group Analyses

Sub-group analyses revealed that DL-based CADx models displayed higher sensitivity on incidentally detected nodules than screening-detected nodules, 0.90 (95% CI 0.77–0.96) *versus* 0.84 (95% CI 0.76–0.90), respectively. This increased reliability in detecting lung cancer came at the cost of specificity with screening-detected nodules having 0.84 (95% CI 0.78–0.89) compared to incidentally detected nodules at 0.70 (95% CI 0.55–0.81). Accounting for threshold effects, screening populations performed better than incidental populations for all risk prediction methods (eFigure 2A–C), particularly clinical risk models: pooled AUC of 0.75 (95% CI 0.69–0.80) in screening-detected nodules *versus* 0.60 (95% CI 0.56–0.64) in incidentally detected nodules (eFigure 2C). The difference between ROC curves for DL-based and physician reader methods *versus* clinical risk models for incidentally detected nodules was particularly pronounced, translating into pooled AUCs of 0.74 (95% CI 0.71–0.77) and 0.77 (95% CI 0.71–0.82) for DL-based models and physician readers, respectively, *versus* 0.60 (95% CI 0.56–0.64) for clinical risk models (eFigure 2).

Further sub-group analyses were carried out on prevalence and geography. For prevalence, the baseline malignancy in CT-detected nodules (4–30 mm) in the US, ~ 5%, [23] was multiplied by a factor of 4, and used as the threshold for classifying a study’s prevalence as high or normal. Thus, datasets with > 20% prevalence were considered high, and < 20%, normal. Further thresholds at 10% and 30% were explored with similar results. For geography, datasets were classified according to continent: Europe, Asia, and North America. Neither prevalence nor geography were found to be a source of heterogeneity.

The analysis was also re-run excluding nodules assessed in follow-up CT scans [24]. The majority of studies assessed nodules from initial CT scans (Table 1). This reduced the pooled sensitivity and specificity of the Lung-RADS-based models, but did not significantly affect any other results.

### Quality Assessment

Overall, a low risk of bias was found in most studies using QUADAS-2 (eTable 4). Selection of participants varied between studies. This may have contributed to biased estimates of sensitivity and specificity as well as inter-study heterogeneity. Therefore, most studies (nine of 17) scored an unclear risk of bias owing to patient selection, but low risk in other categories.

## Discussion

Seventeen studies with external validation data were identified, from which pooled analyses found DL-based models had superior AUC of 0.86 (95% CI 0.83–0.90) as compared to other methods of predicting malignancy in pulmonary nodules (0.83 [95% CI 0.79–0.88] for experienced physician readers and 0.79 [95% CI 0.75–0.83] for clinical risk models). This review attempted to exhaustively search the literature for all studies and models relevant to the research question. There were two common reasons for ineligibility. First, studies did not conduct external validation of the DL-based model being analysed (at final screening, 30 studies had no direct validation against non-DL methods or in an external dataset) (Fig. 1). Second, studies were excluded because they concerned detection of pulmonary nodules, not risk assessment (at final screening, 11 studies with ineligible index tests) (Fig. 1).

In order to evaluate performance across different populations, external validation is crucial [37]. The majority of studies conducted validation on datasets that were used for training or testing (internal validation). In terms of validation against other methods, many studies were validated against other DL-based models, and not models currently used in clinical practice.

For the second commonest exclusion, computer-aided solutions for pulmonary nodule management can be broadly categorised into two types: computer-aided detection (CADe) and CADx (diagnosis) [38]. CADe detects suspicious nodules and segments them for further analysis. CADx provides a nodule- or patient-level classification of the risk of malignancy.

Two previous systematic reviews have studied this issue [39, 40]. Neither, however, directly compared DL-based models with methods used in clinical practice. Nor did they restrict the search to studies that externally validated models in populations other than the population on which they were trained. Only Forte et al. [39] performed a meta-analysis. They considered six studies, five of which are included here [22, 27–29, 35], and one that was excluded due to no external validation. Pooled sensitivity and specificity in Forte et al. [39] were 0.94 (95% CI 0.86–0.98) and 0.69 (95% CI 0.51–0.83), respectively, both with significant heterogeneity. Pooled AUC was 0.90 (95% CI 0.86–0.92) [39]. No quantitative comparisons against physician reader or clinical risk models were performed. The authors noted DL-based models performed well, and that, as non-invasive methods, they could provide support to clinics in diagnosing lung cancer early.

### Limitations

Although these results strongly support the use of externally validated DL-based models, two important limitations were noted. First, only observational studies were found. This was expected given evidentiary requirements for diagnostic tools are not set as high as therapeutic interventions [41].

The second was the high heterogeneity between studies. High heterogeneity is likely given the very different DL-based models under consideration, and the further work required to calibrate some models. Sources of heterogeneity were investigated with sub-group analyses. Neither prevalence nor geography were found to be sources of heterogeneity. Route of detection (screening *versus* incidental) was found to be a potential source for clinical risk models. However, the strongest source of heterogeneity is likely the threshold or operating cut-off point used by researchers in testing the models [39]. The types of thresholds used varied considerably. They included fixing the specificity of models to 0.90 [36], to setting rule-out (definite benignity) malignancy scores (out of 1.0) at 0.05 or rule-in (definite malignancy) malignancy scores to 0.65 [16, 17].

Sensitivity to threshold effects was not investigated due to this variability. Nevertheless, the inclusion of AUC, which captures performance across all possible threshold values, and its concordance with sensitivity and specificity results helped alleviate this concern.

The low risk of bias found in most studies, and no significant publication bias further demonstrate the robustness of the findings.

### Clinical Practice Guidelines

Indeterminate nodules are nodules without obvious signs of benignity (such as calcification) or malignancy (such as spiculation). These nodules are particularly problematic [5, 42]. In order to diagnose cancer or refer high-risk cases for further invasive investigation, clinical risk prediction models are used to aid the physician. At least two pulmonary nodule management guidelines explicitly mention the use of specific clinical risk models. The American College of Chest Physicians (ACCP) recommend using a “validated model” for ≥ 8 mm solid nodules along with or instead of physician judgement [43]. The guideline further notes that the Mayo model is the most validated model for nodules that have been incidentally detected. The British Thoracic Society (BTS) goes further, recommending the Brock model for all nodules ≥ 8 mm in size [44].

Approximately five malignant nodule patients are incidentally detected for every one detected via screening [45]. The evidence for the effectiveness of programme-based management of incidentally detected pulmonary nodules has led to more centres across the US looking to implement them [45]. However, incidental programmes require investment in infrastructure and nodule experts [46]. There is also a concerted drive to increase the uptake of and expand access to low-dose CT screening of at-risk populations for lung cancer [47].

Together these trends raise important challenges. With both early detection programmes detecting more pulmonary nodules, the number of nodules requiring image-based discrimination will cause a surge in workload for healthcare facilities. Clinical risk models, which require manual entry of variables, along with a shortage of nodule experts, mean most health systems are ill-equipped to handle such a surge.[23, 46] DL-based models, however, have the potential to mitigate these challenges by increasing throughput and efficiency, non-invasively [48]. Moreover, their automation can guide and enhance the capabilities of non-experts such as radiographic technologists [49]. By providing reliable, automated analyses of nodules that integrate into radiology workflows, DL-based CADx can assist nodule experts in accurately making faster and more rule-in and rule-out diagnoses [9, 50].

### Future Research

More research needs to be undertaken on how the diagnostic performance of DL-based models translates into improved clinical utility and patient outcomes. Such research should be prospective, and consider a range of settings. While several studies have demonstrated clinical utility [9, 50, 51], further work is needed.

Nodule type and size over time are also important areas for future research. Most studies assessed only the risk of malignancy in initial CT scans. Studies over time on follow-up low-dose CT scans are a future area of research. For nodule type, ground-glass nodules (GGNs) were not considered in any of the datasets analysed. Although GGNs are mostly transient and comprise ~ 2% of nodules, persistent cases tend to have higher malignancy rates (~ 34%) than solid nodules [52]. As such, assessing the malignancy risk of GGNs needs further research.

As DL-based models are calibrated further, and become more routinely used in clinical practice, heterogeneity may reduce, as observed with the Mayo and Brock models for clinical risk [6, 7]. With the potential high-throughput advantages conferred by DL-based models, and their superior or comparable diagnostic performance as compared to other methods, routine clinical use will be important.

## Supplementary Information

Below is the link to the electronic supplementary material.

Supplementary file1 (PDF 451 kb)
