# Supplementary material for: Deep Learning Models for Predicting Malignancy Risk in CT-Detected Pulmonary Nodules: A Systematic Review and Meta-analysis
Source: Lung. 2024 May 23;202(5):625–36. doi: 10.1007/s00408-024-00706-1 (PMC11427562; doi:10.1007/s00408-024-00706-1)
Supplement: Supplementary file 1 — Supplementary file1 (PDF 451 kb) [file 408_2024_706_MOESM1_ESM.pdf]

Supplementary Material

eTable 1. Keyword search terms

Term	Component	Operator
Computer-aided	Index test(s) set #1	AND
Computer aided		OR
Computer-assisted		OR
Computer assisted		OR
CADx		OR
Artificial intelligence		OR
Machine intelligence		OR
Co-learning		OR
Colearning		OR
Machine learning		OR
Deep learning		OR
Predict*	Index test(s) set #2	AND
Diagnos*		OR
Classif*		OR
Estimat*		OR
Evaluat*		OR
Risk		OR
Compute* tomograph*	Index test(s) set #3	AND
Axial tomograph*		OR
CT scan*		OR
CAT scan*		OR
Cancer*	Target condition set #1	AND
Carcinoma*		OR
Neoplas*		OR
Tumour*		OR
Tumor*		OR
Malignan*		OR
Nodule*		OR
Lung*	Target condition set #2	AND
Pulmonary		OR

eFigure 1. Deeks' funnel plot of the included studies and their datasets

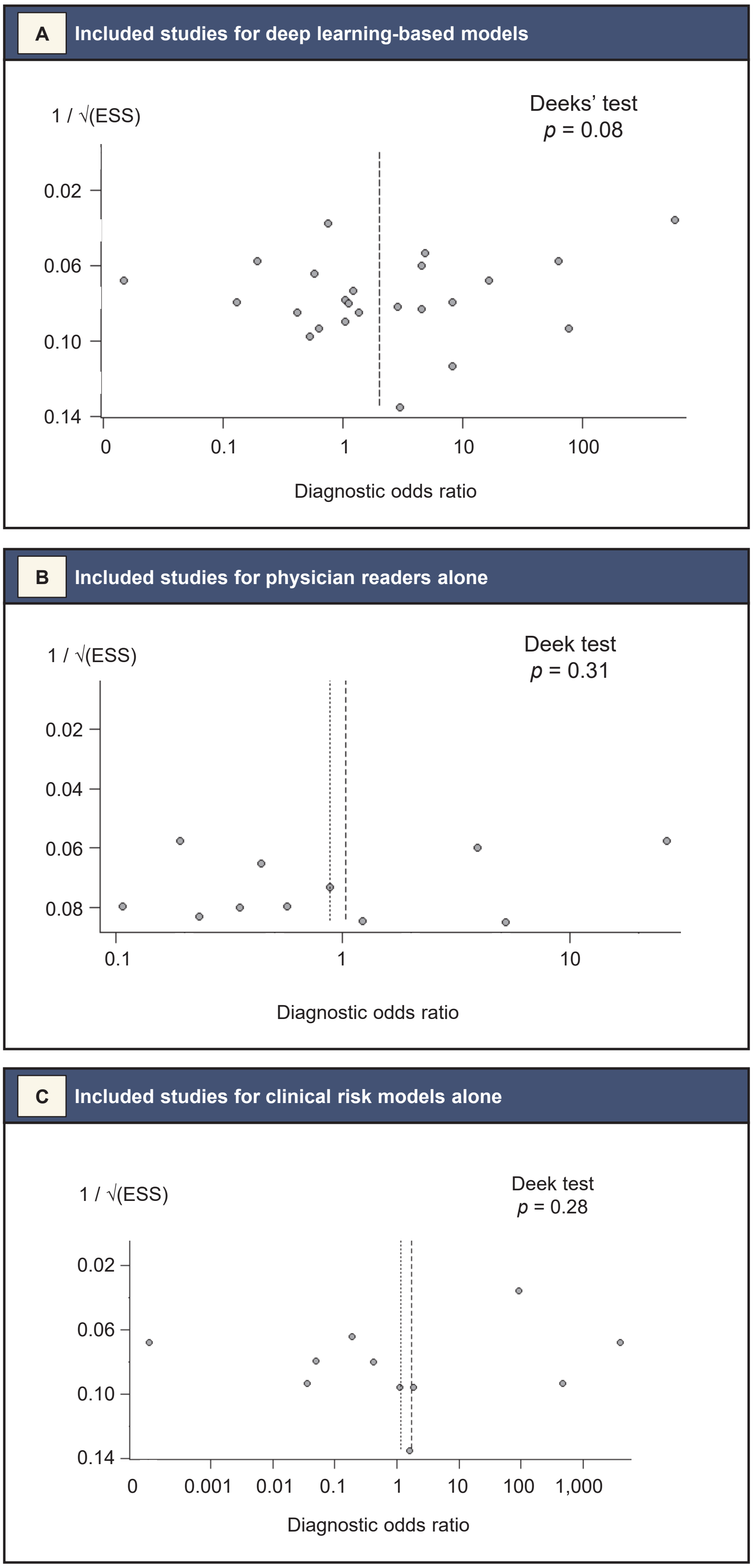

Lung-RADS-based models did not have sufficient data to conduct Deeks' test

ESS, Effective sample size

eTable 2. Selection criteria

Attribute	Inclusion criteria	Exclusion criteria
Study type	• Studies should be in the English language<br>• Studies should be human experimental studies or human observational studies	Studies should not be duplicate studies, case reports & series, non-systematic review articles, non-peer reviewed studies, non-human studies, meeting abstracts & proceedings, nor unpublished
Index test		
Deep learning-based index test	Index test described and investigated should use artificial intelligence (AI) or deep learning (DL) methods – defined as the self-reported use of AI or DL – to classify or otherwise predict the risk of malignancy in pulmonary nodules detected via CT scans	
Study data	External validation of the DL-based index test should be performed on data not used for the initial development or training of the DL-based model	
Direct comparison	DL-based index test should be directly compared with other methods that are in widespread clinical use, the categories of which are:<br>• Physician judgement (radiological image readers)<br>• Clinical risk models: multivariable statistical models that use clinico-demographic (age, smoking history, etc.) or radiological (nodule size, location, etc.) variables as inputs<br>• Lung-RADS-based models: models that allow computers or humans to automatically classify nodules on nodule size, type, and stability over time[1]	
Reference test	Studies should confirm malignancy diagnosis via histopathological (biopsy) within the follow-up period after initial nodule detection	
Target condition and population	Study participants should be ≥18 years old, with at least one solid or part-solid pulmonary nodule (0–30 mm), as identified via CT scan (i.e. studies on ground-glass nodules [GGNs] only are excluded)	
Outcomes	Studies should report at least one of: sensitivity, specificity; areas under the curve (AUC); diagnostic odds ratios; or the number of true-positive, false-negative, true-negative, or false-positive cases (as confirmed by histopathological analysis)	

eFigure 2. ROC curves for screening-detected nodules *versus* incidentally detected nodules

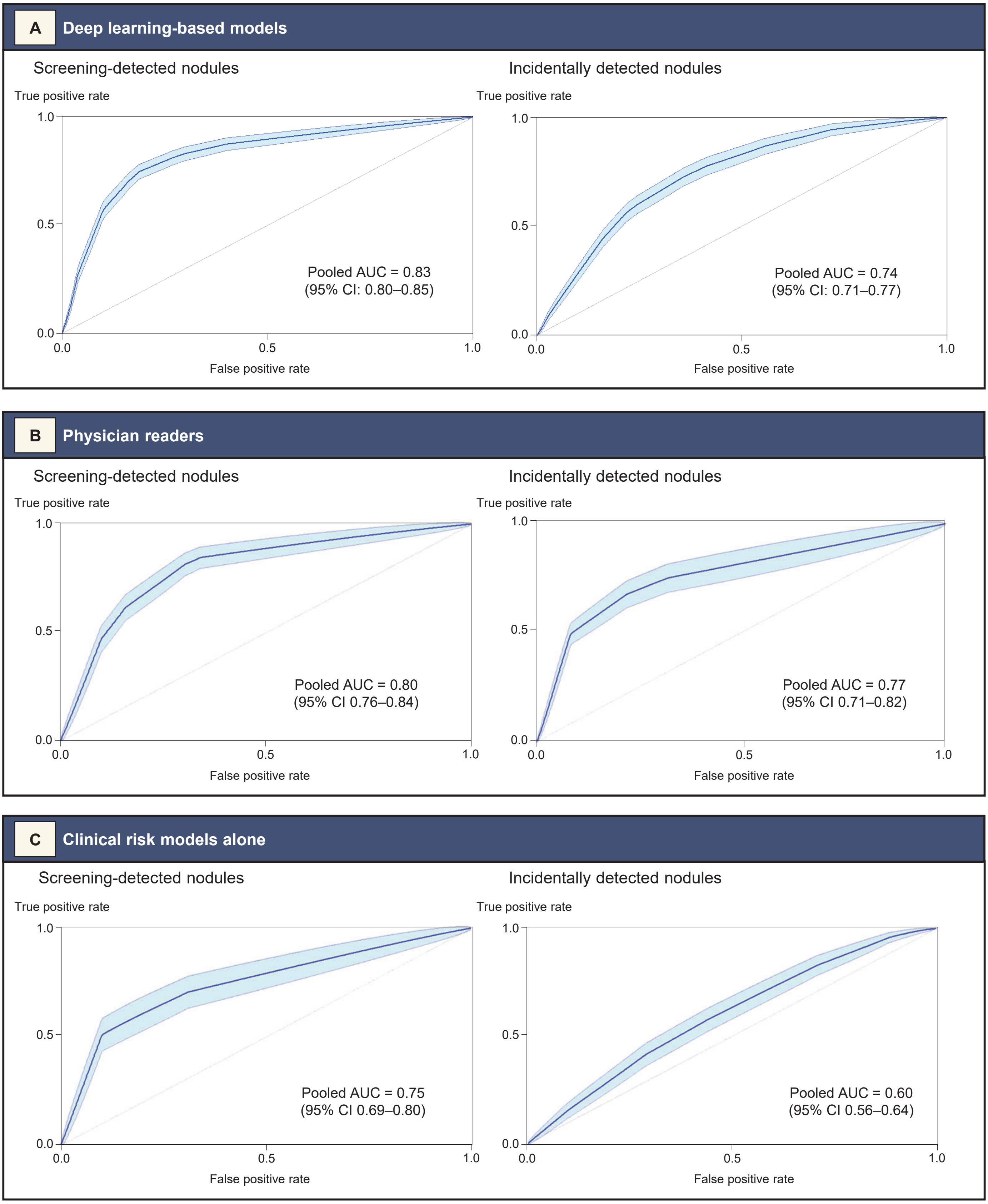

Lung-RADS-based models did not have sufficient data to plot a ROC curve for both routes of detection

eTable 3. Data extracted

Type of data or information	Data and information extracted
Study characteristics	• Author<br>• Publication date<br>• Funding source<br>• Study type<br>• Index test(s)<br>• Reference test
Population characteristics	• Study population country(ies)<br>• Setting<br>• Number of participants in each validation dataset<br>• Number of nodules in each validation dataset<br>• Prevalence of malignancy among participants<br>• Prevalence of malignancy among nodules<br>• Age range<br>• Sex<br>• Participant exclusions<br>• Proportion of smokers (current and former)<br>• Nodule size range<br>• Median nodule size<br>• Nodule type(s)<br>• Route of nodule detection (incidental or screening)
Outcome characteristics	• Outcome(s) reported<br>• Threshold (operating cut-off point)

eTable 4. QUADAS-2 assessment of included studies

ID	Study	Risk of bias				Applicability concerns		
		Patient selection	Index test	Reference standard	Flow and timing	Patient selection	Index test	Reference standard
01	Adams et al 2021	Unclear	Low	Low	Low	Low	Unclear	Low
02	Adams et al 2023	Unclear	Unclear	Low	Low	Low	Low	Low
03	Ardila et al 2019	Low	Unclear	Low	Low	Low	Low	Low
04	Baldwin et al 2020	Low	Low	Low	Low	Low	Low	Low
05	Chen et al 2021	Unclear	Low	Low	Low	Low	Low	Low
06	Chen et al 2022	Unclear	Unclear	Low	Low	Low	Low	Low
07	Çoruh et al 2021	Unclear	Low	Low	Low	Low	Low	Low
08	Gao et al 2021	Low	Low	Low	Low	Low	Low	Low
09	Gao et al 2022	Unclear	Low	Low	Low	Low	Low	Low
10	Huang et al 2019	Unclear	Unclear	Low	Low	Low	Low	Low
11	Hunter et al 2022	Low	Unclear	Low	Low	Unclear	Unclear	Low
12	Jacobs et al 2021	Unclear	Unclear	Low	Low	Low	Low	Low
13	Kim et al 2022	Unclear	Low	Low	Low	Low	Low	Low
14	Liu et al 2020	High	Low	Low	Low	Low	Low	Low
15	Massion et al 2020	Low	Low	Low	Low	Low	Low	Low
16	Trajanovski et al 2021	Low	Low	Low	Low	Low	Low	Low
17	Venkadesh et al 2021	Low	Low	Low	Low	Low	Low	Low

High risk: Unclear risk: Low risk:
